# Supplementary material for: Overall maternal morbidity during pregnancy using the WHO-WOICE tools
Source: PLoS One. 2023 Aug 16;18(8):e0275882. doi: 10.1371/journal.pone.0275882 (PMC10431610; doi:10.1371/journal.pone.0275882)
Supplement: Attachment — Submitted filename: RESPONSES TO COMMENTS.docx [file pone.0275882.s001.docx]

**POINT-BY-POINT RESPONSE TO THE COMMENTS**

Ref : Submission ID 3625416e-5ddf-4751-bacc-e36f8bd91406

| **REVIEWER 1** | |  |
| --- | --- | --- |
|  | **COMMENT** | **RESPONSE** |
| **INTRODUCTION** | 1. The introduction provides a very detailed background on maternal mortality and can be made more concise and streamlined according to the objective. | The introduction has been made more concise ans streamlined according to the objective. |
|  | 2. The last paragraph of the introduction states, “This study will test the feasibility and acceptability of using a tool to measure the impact of pregnancy and childbirth on women's health status. Once validated in our context…”. However, the objective of this study is, “to describe the current state of maternal morbidity in the prefecture of Marrakech.” This study is neither testing the feasibility and acceptability of the tool, nor is it validating it in Morocco. | The objective of this study has been corrected. The new one is : To evaluate the prevalence of nonsevere maternal morbidities (including overall health, domestic and sexual violence, functionality, and mental health) in women during antenatal care in rural versus urban areas.  In order to make the results more concise and streamlined according to the objective, I have tried to present in this article the maternal morbidity in pregnant women. Another article will present the results related to postpartum women. |
| **METHODS** | 1. More detailed description of the study design and sites should be included. For example, which ten health centers were included? How were these centers selected? How many were in rural and how many were in urban areas? | Following your comments, the methods has been revised :   1- This study was conducted in 10 health center in Marrakech Morocco (05 urban and 05 rural). The Inclusion criteria of this health centers are : Representation of the urban and rural areas and the number of prenatal visits exceeds 30 visits per month. 2- A convenience sampling strategy used so that all eligible women invited to participate. Women were invited to participate if they are in their third trimester of pregnancy (28 or more weeks). The exclusion criteria are minors under 18 years of age and women suffering from a mental or physical disability 3- Written informed consent was obtained, except in cases where the woman was illiterate in which case a witness confirmed the accurate reading of the consent form and a thumbprint of the participant was obtained. This procedure was approved by the ethics committees. 4- According to the WHO protocol, for the administration of the questionnaire, each interview lasted between 15 and 30 minutes in total, and the physical examination took between 10 and 15 minutes. 5- This article presents quantitative data. Another article will present the qualitative results of the WHO-WOICE feasibility and acceptability study. |
|  | 2. What was the sampling technique used to recruit participants in the study? |  |
|  | 3. Please mention details about the informed consent process. |  |
|  | 4. It is mentioned in the first paragraph that, “the time allocated to the questionnaire (45 to 65 minutes)…”. The second paragraph says that, “each interview with the woman lasted between 15 and 30 minutes in total.” What was the reason that interviews only took about half the suggested time? |  |
|  | 5. “The data analysis was done with the two software programs Epi Info 7 for the quantitative data and Invivo 10 was used for the qualitative data.” What kind of qualitative data were included? How were the quantitative and qualitative data analyzed? |  |
| **RESULTS** | 1. When comparing the proportion of a characteristic between two groups (for example, anxiety and depression in women during PNC and PoNC), the statistical significance should be reported. | The statistical significance has been reported (Table N°3) |
|  | 2. Tables 1 and 2 should be combined. | Table 1 and 2 has been combined |
|  | 3. The tables are not referenced in the text which is a little difficult to follow. | In this version, all the tables are referenced in the text |
|  | 4. “Nevertheless, 39.29% in PNC and 37.94% in PoNC declared….”. Table IV says that 44.75% women in the PNC consultation declared that their health status was neither good nor bad. | This sentence has been corrected |
|  | 5. “Using both tools, it was revealed that PNC women were more likely to suffer from anxiety (83.65% vs. 29.24% in PNC) and also signs of depression (43% vs. 17.78% in PNC).” It is unclear what the percentages in the parentheses represent. Additionally, what were the signs of depression? Table V: Mental health only describes symptoms of anxiety and not depression. | Percentages in the parentheses are for prenatal versus postnatal women.  In the corrected article, only the anxiety and depression scores for pregnant women were presented |
|  | 6. In table V: Mental health, what does the no and yes below item no. 7 on the anxiety score refer to? | It was an error |
|  | 7. In table VII, the numbers in the row “number of complications diagnosed” are not equal to the number of women who received prenatal consultation for both urban and rural women. There is a similar issue with the “number of complications diagnosed” for urban women in the postnatal consultation. There appears to be some missing data which should be mentioned. | Yes just some missing data  **In order to make the article easy to read, I have presented the demographic characteristics and clinical conditions without mentioning the rural/urban distribution. Also, I have presented just the results for pregnant women.** |
|  | 8. In table VII, the numbers in the subcategories of types of indirect complications in the PNC rural column equal to 55, when there were 59 women who experienced indirect complications. Similarly, the direct complications in the same column equal to 33, when there were 58 women who experienced direct complications. | The reasons for this difference in numbers are:  - Some women may have one or more complications  - Some women can have direct and indirect complications  - In addition, I have presented in the article the main direct or indirect complications. Other complications not listed in the manuscrit.  **Some Errors have been corrected** |
|  | 9. In table VII, there are 32 women with back pain in the PNC urban column, when there were 28 women in this group who experienced indirect complications. | Yes just some missing data |
|  | 10. Some of the headings in table VII are in French. | This sentence has been corrected |
|  | 11. The results do not show the findings from the qualitative part of the study. | The qualitative results will be the subject of another article |
| **DISCUSSION** | 1. The recommendations are considerably longer than the discussion. The recommendation should be shortened and included within the discussion section, keeping in view the PLOS ONE format for a research article. | The recommandations are included within the discussion. |
|  | 2. “In addition, our study found that the prevalence of emotional abuse was more frequent than physical abuse.” This finding is not shown in the results section. | This version of the manuscit no longer includes this sentence. |
|  | 3. “This result does not corroborate with a study conducted in Egypt in 2017 [18], which showed that women in rural areas are more exposed to domestic violence than those in urban areas.” The results state that, “…with rates varying by setting from 12% in urban areas to 29% in rural areas.” This indicates that the findings of the present study do corroborate with the study conducted in Egypt. | The prevalence of domestic violence between urban and rural areas is not included in this version of the manuscrit. |
|  | 4. Some of the recommendations are beyond the scope of this study. For example, the impact of seeing pregnancy as an opportunity, adopting a rights-based approach, and premarital consultation were not evaluated in the study. Therefore, these recommendations cannot be directly derived from the results of this study. | Following your comments, the recommendations has been revised |
|  | 5. It would be good to comment on the generalizability of the findings of this study. | The generalizability of the findings of this study has been included into the discussion. |
| **CONCLUSION** | 1. Overall, the conclusion seems generic and not specific to the salient findings of the study and their broader implications. | Following your comments, the conclusion has been revised |
|  | 2. Typically, there are no references in the conclusion. |  |
|  | 3. “Although women interviewed report a high level of satisfaction with MM tools…”. This statement is not supported by this study. |  |
