# Supplementary material for: Overall maternal morbidity during pregnancy using the WHO-WOICE tools
Source: PLoS One. 2023 Aug 16;18(8):e0275882. doi: 10.1371/journal.pone.0275882 (PMC10431610; doi:10.1371/journal.pone.0275882)
Supplement: Attachment — Submitted filename: RESPONSES TO COMMENTS 2.docx [file pone.0275882.s002.docx]

**POINT-BY-POINT RESPONSE TO THE COMMENTS**

**Overall Maternal Morbidity during Pregnancy using the WHO-WOICE tools**

**PLOS ONE**

| **REVIEWER 1** | |  |
| --- | --- | --- |
|  | **COMMENT** | **RESPONSE** |
| **INTRODUCTION** | 1) authors described in their methods that they have applied the WOICE tool, regarding functioning, however I could not find the description of the results. Consider including it. | The WOICE tool is made up of several sections. This article focuses on results from the prenatal period. |
|  | 2) In table 3, authors included sexual satisfaction in French, correct it. | Already corrected |
| **METHODS** | 3) Why did authors choose to compare rural and urban settings for anxiety, depression and sexual satisfaction, but not for the other variables included in tables 1 and 2? Presentation of results must be uniform throughout the article. Discuss the best way to present results. I believe that the comparison between both settings is interesting, and if authors decide to do it, they should uniformize the analysis in the other tables. | The rural/urban comparison has been added to table 2 and the discussion. Table 1 presents the general characteristics, and the authors do not consider it relevant to add data from both rural and urban areas. |
